# The Reliability of a Modified Kalamazoo Consensus Statement Checklist for Assessing the Communication Skills of Anesthesia Doctors in the Simulated Environment

**DOI:** 10.7759/cureus.77640

**Published:** 2025-01-18

**Authors:** Yograjan Ramalingam, Aishwarya PM, Lakshmi Nagalingam, Krishna Prasanth, Ashni Bhandari

**Affiliations:** 1 Anaesthesiology, Sree Balaji Medical College and Hospital, Chennai, IND; 2 Community Medicine, Sree Balaji Medical College and Hospital, Chennai, IND; 3 Biostatistics and Epidemiology, Sree Balaji Medical College and Hospital, Chennai, IND

**Keywords:** communication skills, healthcare quality improvement, modified kalamazoo consensus statement, multidisciplinary training, patient safety, simulation-based education

## Abstract

Background

Clear communication is vital in anesthesiology to ensure patient safety and deliver high-quality care. Communication errors remain a significant cause of medical errors worldwide. The Modified Kalamazoo Consensus Statement Checklist (MKCSC) offers a structured approach to assessing communication skills. This study evaluates the reliability, validity, and applicability of the MKCSC in India’s diverse, multidisciplinary healthcare setting, focusing on its impact on improving communication and patient care through the Program for the Approach to Complex Encounters (PACE).

Aim

This study aimed to assess the effectiveness of the MKCSC in evaluating and enhancing communication skills among physicians, resident doctors, and nurses and to examine the role of PACE in improving communication outcomes.

Methods

A repeated cross-sectional study was conducted at Dr. Rela Institute and Medical Center, Chennai, involving 200 participants, including doctors (≥2 years post-MD), resident doctors, and nurses. Participants underwent pre-intervention simulations assessed using the MKCSC, followed by PACE training. Communication scores were evaluated at one-month and five-month follow-ups using paired t-tests, intra-class correlation coefficients (ICCs) for interrater reliability, and Cronbach’s alpha for internal consistency.

Results

The MKCSC demonstrated excellent internal consistency (Cronbach’s alpha: 0.89-0.91) and interrater reliability (ICC: 0.85-0.88). Communication scores improved significantly from 23.5 ± 1.4 pre-intervention to 28.9 ± 2.1 at one month and 30.5 ± 2.2 at five months (p < 0.001). Improvements were consistent across professional groups, with significant gains in building relationships and understanding perspectives.

Conclusion

The MKCSC is a reliable and adaptable tool for assessing communication skills. Combined with PACE, it fosters empathy, enhances teamwork, and bridges communication gaps, contributing to improved patient safety. Further research is needed to validate its use in real-world clinical settings and expand its impact on healthcare delivery.

## Introduction

Good communication is crucial for safe and quality patient care, especially in anesthesiology, where quick and accurate decisions can make a huge difference in patient outcomes [1]. In high-pressure situations like these, clear communication among healthcare professionals helps avoid misunderstandings that could lead to mistakes or delays in treatment [2,3]. When teams communicate well, they keep patients safer, and they also create a more supportive and collaborative environment for everyone involved [4]. However, when communication breaks down, it can lead to serious problems like errors, delays, and teamwork struggles [2,5]. Hence, the focus is on developing tools to assess and improve communication skills in medical training and practice.

The Kalamazoo Consensus Statement, introduced in 1999, focuses on seven key elements of good communication: building relationships, starting conversations, gathering information, understanding others' perspectives, sharing information, finding common ground, and bringing discussions to a close. These ideas were later used to create the Modified Kalamazoo Consensus Statement Checklist (MKCSC), which is tailored specifically for healthcare teams, including doctors, residents, and nurses [2,5].

The checklist improves teamwork and understanding among healthcare professionals, which is critical when patient care depends on group effort. The MKCSC gives teams a clear approach to communication, promoting better connections and collaboration. Ultimately, this strengthens teams and improves patient care [3,6].

Communication tools such as the MKCSC have shown their effectiveness globally by enhancing patient safety, reducing errors, and improving teamwork [2,7]. These tools offer healthcare teams a structured way to communicate, which is essential in fast-paced environments. On top of that, simulation-based training gives professionals a chance to practice their communication skills in a safe space, with feedback to help them get better over time [8,9].

Although tools like the MKCSC have shown good results in countries like the US and parts of Europe, they have not been widely adopted in lower- and middle-income nations, such as India [6,2]. India’s healthcare system deals with unique obstacles, such as a large number of patients per provider, limited resources, and a diverse population with different needs. These factors can make communication among healthcare workers more difficult. This underscores the importance of having tailored training and assessment tools that can address the specific challenges faced by healthcare teams in these regions [2,9,10].

Adapting the MKCSC to the Indian context

Doctors, resident physicians, and nurses provided feedback for the MKCSC tool to adapt to India’s healthcare system to make sure it was practical and culturally appropriate [8,9,10].

To make sure it worked well, a thorough process was followed. Experts validated the content, and the tool was tested in both simulated environments and real clinical settings to check its reliability and accuracy [8,11,12].

Once the tool was ready (Appendix 1), it was rolled out along with training programs for healthcare professionals. These programs covered both the basics of good communication and how to use the tool in day-to-day situations [2,13]. The aim was to ensure healthcare workers could use the tool effectively to improve patient care.

The training included hands-on exercises like role-playing and feedback sessions to help people really grasp the communication skills needed [14,15]. Ongoing monitoring will be important to see how the tool impacts patient outcomes, team dynamics, and overall satisfaction. This will help us make sure it is working well and improving over time [16].

## Materials and methods

Study design and objective

This observational study assessed communication skills prior to training, with follow-up evaluations conducted at one-month and five-month intervals using the MKCSC checklist. The objective was to determine whether the Program for the Approach to Complex Encounters (PACE) intervention could produce lasting improvements in communication among healthcare teams in a simulated environment for patients who had undergone elective surgery for the first time in the Indian context.

Tool development

The MKCSC, originally developed for Western healthcare systems, was adapted to suit the Indian healthcare environment. Modifications were informed by consultations with physicians, nurses, and hospital staff to ensure cultural and contextual relevance. Language and examples were localized, and hierarchical team structures common in Indian hospitals were considered. These adjustments preserved the tool's foundational principles, such as active listening and effective information exchange, while aligning with the unique dynamics of Indian healthcare teams.

Tool implementation

Simulations designed to reflect real-world scenarios encountered by healthcare professionals were conducted using the updated MKCSC, which was validated by one clinical psychologist, three anesthesiologists, one epidemiologist, and one doctor of preventive medicine [17]. Scenarios included preoperative discussions, crisis management during procedures, and handovers between teams. Trained actors portrayed patients, and the simulation environments were constructed to replicate clinical realities, enabling participants to engage in authentic interactions.

Participant selection

The study included 200 participants: 80 experienced physicians, 60 resident doctors undergoing postgraduate training, and 60 nurses. All participants were selected from teaching hospitals to ensure a representative mix of roles and to emphasize the importance of interprofessional teamwork in the Indian healthcare setting.

PACE intervention

The PACE intervention was implemented as a structured training program designed to enhance communication skills. The program lasted for five months and incorporated didactic sessions, role-playing exercises, and feedback, including video-based self-assessments [18,19]. Each participant had undergone four didactic sessions of two hours each, three role-playing exercises (one for breaking bad news, one for conflict resolution, one for de-escalating angry patients) of 30 minutes each, and two self-assessment video sessions of one hour each. All enrolled participants completed the training with a zero dropout rate. This approach was aimed at providing participants with practical, actionable skills applicable to their daily clinical practice.

Tool training

Raters included subject matter experts and peer observers who were trained on the proper use of the MKCSC. Training sessions covered scoring methodology, included practice sessions with example scenarios and were followed by group discussions to ensure consistency in scoring criteria and alignment among raters.

Scoring

The MKCSC evaluated communication across seven domains: building relationships, initiating conversations, gathering information, understanding perspectives, sharing information, reaching agreements, and closing conversations. Each domain was rated on a five-point Likert scale, with additional sections for narrative feedback highlighting strengths and areas for improvement [10].

Data collection and analysis

Baseline communication scores were collected by four people who were not part of the study and were given training on effective data collection before the intervention. The individuals were not blinded, and the tools were filled by healthcare professionals and patients who had elective surgery and were asked to help as actors for the simulation scenarios. Post-intervention assessments were conducted at one-month and five-month intervals to monitor progress. Reliability was evaluated using Cronbach's alpha (range: 0.89-0.91), while interrater and intrarater consistency were assessed using intra-class correlation coefficients (ICC range: 0.85-0.88) [10]. The MKCSC utilized a five-point Likert scale, ranging from one (poor) to five (excellent), to evaluate communication skills across seven domains. This scale provided a structured and standardized approach to capturing both quantitative and qualitative aspects of communication performance [10]. Changes in scores were analyzed using paired t-tests, with subgroup comparisons between physicians, resident doctors, and nurses. Statistical analyses were performed using SPSS (IBM SPSS Statistics for Windows, IBM Corp., Version 27, Armonk, NY), with a significance level set at p < 0.05.

Ethical considerations

The study was approved by the institutional ethics committees of participating hospitals. Written informed consent was obtained from all participants, ensuring voluntary participation. Simulations were recorded for evaluation purposes, with all data anonymized to safeguard confidentiality.

## Results

Participant demographics

**Figure 1:**
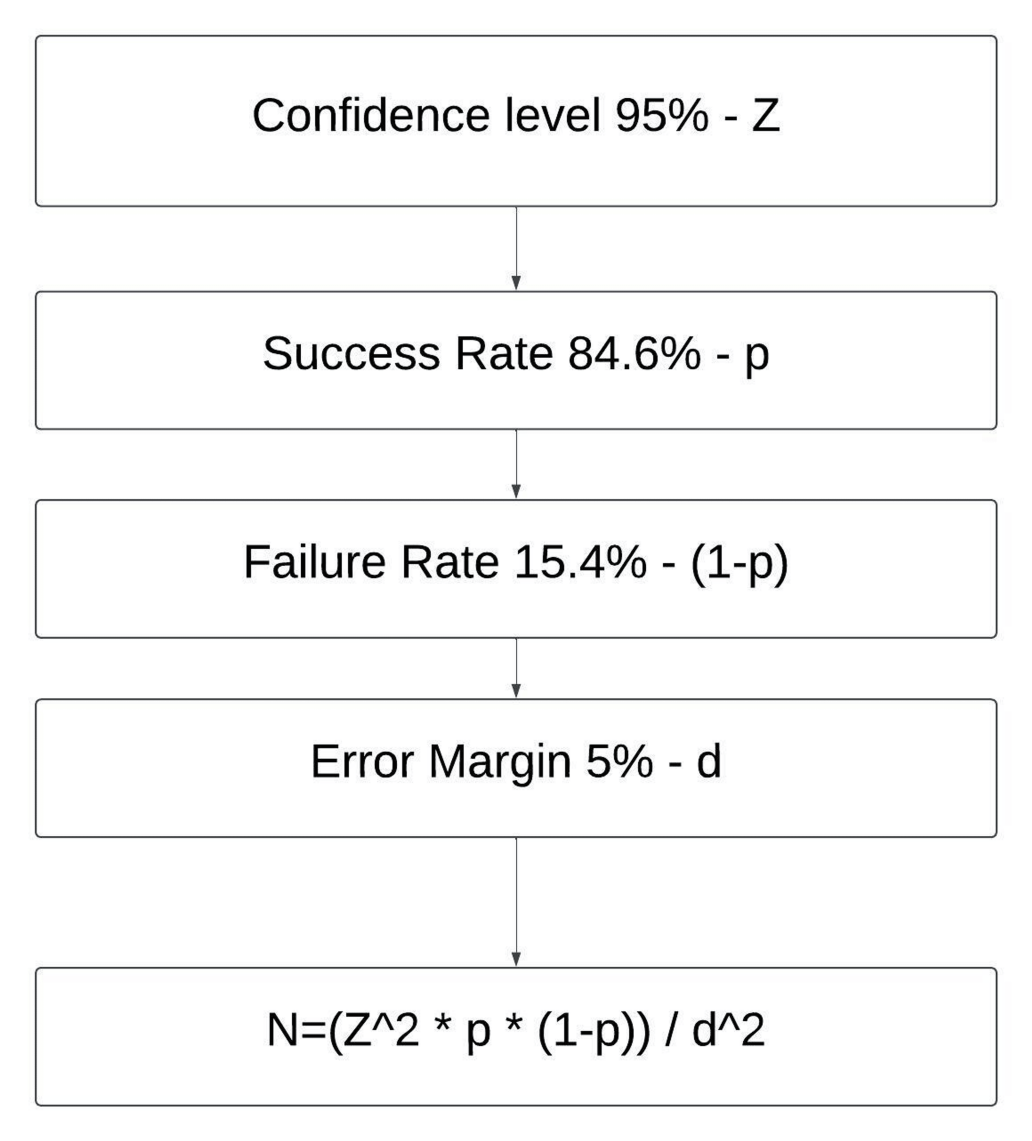
Sample size selection using single proportion sample size formula

A sample size of 200 was calculated using the single proportion sample size formula (Appendix 2) with a confidence level of 95% and an error margin of 5%, as shown in Figure 1. This study included 200 participants, consisting of 80 physicians with a minimum of two years of post-MD experience, 60 resident doctors in postgraduate training, and 60 nurses, including bedside nurses and nurse administrators. A detailed summary of demographic data is provided in Table 1.

**Table 1:** Participant demographics

Professional group	n	Mean age (years)	Gender (male:female)	Mean experience (years)
Physicians	80	35 ± 4.1	45:35:00	8.3 ± 2.2
Resident Doctors	60	28 ± 2.5	32:28:00	2.1 ± 0.9
Nurses	60	31 ± 3.8	15:45	6.5 ± 1.8
Total	200	-	93:48:00	-

Pre-test scores

Baseline communication scores, assessed using the MKCSC, revealed moderate proficiency across the seven communication domains. Scores ranged from 3.2 to 3.6, with a mean score of 3.4 ± 0.2 on a five-point scale. These findings underscore substantial opportunities for enhancement in communication skills across all professional groups.

Post-test scores at one month

One month following the PACE intervention, participants exhibited significant improvements in communication scores across all domains. The overall mean score increased to 4.1 ± 0.3 (p < 0.001). Improvements were observed consistently across physicians, resident doctors, and nurses, with the most pronounced gains in the domains of understanding perspectives and building relationships.

Post-test scores at five months

At the five-month follow-up, the enhancements in communication skills were sustained, with an overall mean score of 4.3 ± 0.2 (p < 0.001 compared to pre-test). Scores across all seven domains remained higher than pre-test levels, indicating the enduring effectiveness of the PACE intervention.

Interrater and intrarater reliability

The ICC for interrater reliability was excellent, with an ICC value of 0.85 across all domains. Intrarater reliability, measured by Cronbach’s alpha, ranged from 0.88 to 0.91, demonstrating strong internal consistency of the MKCSC over time.

Domain-specific performance

The average scores across the seven communication domains at each time point are presented in Table 2. Notably, the domains of gathering information and sharing information exhibited the highest percentage improvements, reflecting enhanced participant capabilities in information exchange and interdisciplinary collaboration.

**Table 2:** Communication scores across time points

Communication domain	Pre-test (mean ± SD)	One-month post-test (mean ± SD)	Five-month post-test (mean ± SD)
Building relationships	3.4 ± 0.3	4.2 ± 0.3	4.4 ± 0.2
Opening discussions	3.3 ± 0.4	4.0 ± 0.3	4.2 ± 0.3
Gathering information	3.5 ± 0.3	4.3 ± 0.3	4.5 ± 0.2
Understanding perspectives	3.2 ± 0.4	4.1 ± 0.4	4.4 ± 0.3
Sharing information	3.4 ± 0.3	4.0 ± 0.3	4.3 ± 0.3
Reaching agreement	3.3 ± 0.3	4.2 ± 0.3	4.4 ± 0.2
Providing closure	3.4 ± 0.3	4.1 ± 0.3	4.3 ± 0.3

Key findings

The PACE program demonstrated its effectiveness in enhancing communication skills, with post-intervention scores showing statistically significant improvements at both the one-month and five-month follow-ups (p < 0.001). The sustained gains observed across all communication domains at the five-month mark confirm the lasting impact of the intervention [20]. Additionally, the MKCSC exhibited high reliability across all time points, underscoring its consistency and applicability within the context of Indian healthcare settings.

## Discussion

Findings

The findings from this study highlight the reliability and validity of the MKCSC as an important tool for assessing and enhancing communication skills among healthcare professionals in a simulated environment [8,5]. The study’s results highlight the checklist’s adaptability in the Indian healthcare context, showcasing its potential to drive meaningful and sustainable improvements in communication proficiency through structured interventions such as the PACE [1,3].

Consistent with global standards, the MKCSC showed high interrater and intrarater reliability, with scores remaining stable across all professional categories [2,21]. The improvements in communication performance observed at the one- and five-month post-tests confirm the intervention’s effectiveness in identifying communication gaps and fostering skill enhancement [12,22]. These findings also align with international studies emphasizing the utility of standardized tools in promoting team-based communication and minimizing errors in high-stakes clinical settings [3,11,19].

Impact of the PACE intervention

The significant gains observed in communication scores highlight the effectiveness of the PACE intervention in consolidating and reinforcing learned skills [10,7]. Structured programs that combine simulation-based exercises, role-playing scenarios, and iterative feedback loops bridge the gap between theoretical knowledge and its practical application [8,7,23]. The intervention’s success in the Indian context indicates the feasibility of scaling similar initiatives to address broader communication challenges in resource-constrained healthcare environments [4,22,24].

A key strength of this study is its focus on multidisciplinary teams, reflecting the complexity of communication in modern healthcare settings. The MKCSC proved to be a versatile tool, effectively capturing communication dynamics across physicians, resident doctors, and nurses. This is particularly relevant in the Indian healthcare context, where hierarchical structures and cultural diversity often pose challenges to seamless communication. A common language for assessing and improving communication fosters a culture of collaboration and mutual respect, bridging the disciplinary divide and ultimately improving patient outcomes [25,26].

The sustained gains observed at the five-month follow-up are a testament to the durability of the intervention’s impact. These findings underscore the importance of structured follow-ups in reinforcing learned skills and ensuring their integration into daily clinical practice. However, the lack of longer-term data beyond five months limits the ability to fully evaluate the intervention’s durability. Future studies should aim to extend follow-up periods to provide a more comprehensive understanding of the tool’s long-term effectiveness.

Limitations and future directions

The reliance on a simulated environment, while ensuring consistency and reproducibility, may not be in alignment with the complexities of a live clinical setting. Simulations, although effective for building foundational skills, cannot fully capture the nuances of live patient interactions and dynamic team communication.

The study’s five-month follow-up showcases the durability of the intervention’s impact over the medium term. However, future research should focus on and explore longer-term outcomes to evaluate whether these gains persist beyond the study period. Using the MKCSC in different healthcare environments, including rural and low-resource areas, could give us a better understanding of how well it works in various settings and if it can be used more widely.

In addition to this, integrating advanced technologies such as digital platforms and AI-driven analytics into the MKCSC checklist framework could improve the precision and efficiency of communication training and assessment [18]. Future studies and research should analyze how the MKCSC checklist affects patient satisfaction and clinical results to better confirm how useful it is.

To maximize its utility, the MKCSC should be evaluated in diverse healthcare settings, including rural and resource-limited environments, where communication challenges may differ from those in urban, tertiary care hospitals. Incorporating digital tools and artificial intelligence into the MKCSC framework could further enhance its precision, scalability, and adaptability. Additionally, incorporating the checklist into routine clinical workflows and linking its use to measurable patient outcomes could provide valuable insights into its real-world applicability. Future studies should also explore the tool’s potential in fostering patient-centered communication, assessing its impact on metrics such as patient satisfaction and safety.

## Conclusions

This study provides solid evidence supporting the MKCSC as a reliable and adaptable tool for evaluating and improving communication skills among healthcare professionals in India. Structured programs like PACE provide an effective way to tackle communication issues, improve teamwork, and enhance patient safety. Ongoing research, especially in real-world clinical environments, will be crucial to fully realize the checklist’s potential and promote its broader use across healthcare systems.

## Appendices

Appendix 1

Modified Kalamazoo Consensus Statement Checklist in Tamil

அனஸ்தீசியா தொடர்பான தொடர்புக் குறியீடு மதிப்பீடு:

மதிப்பீட்டு அளவுகோல்:

1 - மோசம்

2 - சராசரி

3 - நல்லது

4 - மிகவும் நல்லது

5 - சிறந்தது

மதிப்பீட்டு பகுதிகள்:

தொடர்பு உருவாக்குதல்:

1.    நோயாளி மற்றும் குடும்பத்தினரிடம் அன்பான முறையில் வாழ்த்துக்கள் தெரிவித்து கவலை வெளிப்படுத்துதல்.

2.    எளிமையான மொழி மற்றும் உடல்மொழியைப் பயன்படுத்தி பரிவை வெளிப்படுத்துதல்.

3.    அவர்களின் உணர்வுகளை மதித்து, உரையாடலில் ஈடுபடுதல்.

விவாதத்தை தொடங்குதல்:

1.    குறுக்கிடாமல் நோயாளியின் ஆரம்ப சிந்தனைகளை கேட்க அனுமதித்தல்.

2.    கேள்விகளை முன்வைத்து அனைத்து கவலைகளையும் தெளிவுபடுத்துதல்.

3.    ஆலோசனைகளை தெளிவாக விளக்குதல்.

தகவல் சேகரித்தல்:

1.    கேள்விகள் மூலம் முழுமையான மருத்துவ மற்றும் தனிப்பட்ட பின்னணி சேகரித்தல்.

2.    தேவைப்பட்ட இடங்களில்  கேள்விகளால் குறிப்புகளை உறுதிசெய்தல்.

3.    நோயாளியின் தகவல்களை சரிபார்க்க சுருக்கமாக மறுபரிசீலனை செய்தல்.

4.    நோயாளி மற்றும் குடும்பத்தினரின் எண்ணங்களைப்

புரிந்துகொள்ளுதல்:

1.    அவர்களின் அனுபவத்தைப் பாதிக்கும் வெளிப்புற காரணிகளை ஆராய்தல்.

2.    கருத்துக்களையும் எதிர்பார்ப்புகளையும் கேட்டு, அச்சங்கள் அல்லது தவறான புரிதல்களை சரிசெய்தல்.

தகவல் பகிருதல்:

1.    அனஸ்தீசியா செயல்முறைகள் குறித்து புரிதல் நிலையை மதிப்பீடு செய்தல்.

2.    சிக்கல்களையும் விளக்கங்களையும் எளிய மொழியில் எடுத்துக்கூறுதல்.

3.    கேள்விகளை ஊக்குவித்து தெளிவுபடுத்துதல்.

ஒப்பந்தத்தை அடைவது:

1.    அனஸ்தீசியா திட்டங்களைப் பற்றி நோயாளி மற்றும் குடும்பத்தை உட்படுத்துதல்.

2.    பரஸ்பர புரிதலுக்கான திட்டத்தை உறுதிசெய்தல்.

3.    தேவையாயின் மாற்று ஆதாரங்களைச் சொல்லித்தருதல்.

முடிவு செய்தல்:

1.    அனைத்து கேள்விகளையும் கவலைகளையும் தீர்க்க உறுதிசெய்தல்.

2.    முக்கிய அம்சங்களை சுருக்கி, அடுத்த கட்டங்களை விளக்குதல்.

3.    பின்தொடர்ச்சிக்கான தகவல் தொடர்பு விவரங்களை வழங்குதல்.

பரிவை வெளிப்படுத்துதல்:

1.    தகுந்த சாந்த மனோபாவத்துடன் உறுதியாக நடந்து கொள்ளுதல்.

2.    உணர்ச்சிபூர்வமான எண்ணங்களுக்கு தகுந்த ஆதரவு அளித்தல்.

தகவல்களை தெளிவாக வெளிப்படுத்துதல்:

1.    அனஸ்தீசியாவின் தீவிரத்தையும் சாதாரண செயல்முறையையும் தெளிவாக விளக்குதல்.

2.    நோயாளியின் பராமரிப்பில் ஈடுபட்டுள்ள மற்ற மருத்துவர்களின் உள்ளீட்டை கருத்தில் கொள்ளுதல்

3.    மயக்க மருந்திலிருந்து மீண்டு வருவதற்கான போக்கை கோடிட்டு காட்டுதல்

4.    கவனிப்பு விவரங்களை தெளிவு படுத்துதல்

Modified Kalamazoo Consensus Statement Checklist in English

Anesthesia Communication Skills Assessment

Rating Scale:

1 - Poor 2 - Fair 3 - Good 4 - Very Good 5 - Excellent 

Assessment Areas:

1. Builds a Relationship:

Greets patient and family, expressing genuine interest and concern.

Uses compassionate language and body language to convey empathy.

Engages with the patient and family, acknowledging their feelings and statements.

2. Opens the Discussion:

Allows the patient and family to express initial concerns without interruption.

Uses open-ended questions to ensure all anesthesia-related concerns are addressed.

Clearly explains the agenda for the anesthesia consultation.

3. Gathers Information:

Uses open-ended questions to gather comprehensive medical and personal history relevant to anesthesia.

Clarifies specific details using closed questions when necessary.

Summarizes information back to the patient and family to confirm accuracy.

4. Understands Patient and Family Perspective:

Inquires about external factors that might influence anesthesia experience or decisions.

Elicits beliefs and expectations regarding anesthesia, addressing any myths or concerns.

5. Shares Information:

Gauges patient/family understanding of anesthesia procedures and options.

Explains the anesthesia process and potential risks in comprehensible terms.

Encourages questions to ensure clarity and understanding.

6. Reaches Agreement:

Involves patient and family in decisions regarding anesthesia plans.

Confirms mutual understanding of the planned anesthesia approach and contingencies.

Discusses alternative resources or information if required.

7. Provides Closure:

Ensures all questions or concerns about anesthesia are addressed.

Summarizes key points and verifies understanding of next steps.

Provides contact information for any follow-up questions and establishes follow-up timelines.

8. Demonstrates Empathy:

Maintains an appropriate and reassuring demeanor throughout anesthesia discussions.

Recognizes and validates emotional responses concerning anesthesia fears.

Responds to emotional cues with appropriate reassurance or support.

9. Communicates Accurate Information:

Clearly communicates the seriousness and routine nature of anesthesia in relation to the procedure.

Considers input from other clinicians involved in the patient's care.

Outlines the expected course of recovery from anesthesia and presents care options clearly to enable informed decision-making.

Appendix 2

Formula for Calculation of Single Proportion Sample Size

$N=(Z^{2}*p*(1-p))/d^{2}$

where:

N - Required sample size Z - 95% confidence level p - 84.7% - proportion observed from literature (1-p) - 15.3% d - 5% - expected difference in error margin
